# Preparation and Application of a Hydrochar-Based Palladium Nanocatalyst for the Reduction of Nitroarenes

**DOI:** 10.3390/molecules26226859

**Published:** 2021-11-13

**Authors:** Melike Çalışkan, Sema Akay, Berkant Kayan, Talat Baran, Dimitrios Kalderis

**Affiliations:** 1 Department of Chemistry, Faculty of Science and Letters, Aksaray University, Aksaray 68100, Turkey; melikecaliskan.61@gmail.com (M.Ç.); sema.akay7@gmail.com (S.A.); berkantkayan@gmail.com (B.K.); talatbaran_66@hotmail.com (T.B.); 2 Department of Electronic Engineering, Hellenic Mediterranean University, 73100 Chania, Greece

**Keywords:** coffee waste, hydrochar, nitroarenes, catalytic reduction, nanocatalyst, palladium

## Abstract

In the present study, a novel heterogeneous catalyst was successfully fabricated through the decoration of palladium nanoparticles on the surface of designed Fe_3_O_4_-coffee waste composite (Pd-Fe_3_O_4_-CWH) for the catalytic reduction of nitroarenes. Various characterization techniques such as XRD, FE-SEM and EDS were used to establish its nano-sized chemical structure. It was determined that Pd-Fe_3_O_4_-CWH is a useful nanocatalyst, which can efficiently reduce various nitroarenes, including 4-nitrobenzoic acid (4-NBA), 4-nitroaniline (4-NA), 4-nitro-o-phenylenediamine (4-NPD), 2-nitroaniline (2-NA) and 3-nitroanisole (3-NAS), using NaBH_4_ in aqueous media and ambient conditions. Catalytic reactions were monitored with the help of high-performance liquid chromatography. Additionally, Pd-Fe_3_O_4_-CWH was proved to be a reusable catalyst by maintaining its catalytic activity through six successive runs. Moreover, the nanocatalyst displayed a superior catalytic performance compared to other catalysts by providing a shorter reaction time to complete the reduction in nitroarenes.

## 1. Introduction

Due to the increasing demand for sustainable recovery and the exploitation of biowaste, the conversion of biowaste into valuable products has attracted a lot of attention. At the same time, the atmospheric emissions from the use of fossil fuels cause problems such as global warming, climate change and environmental pollution. Furthermore, fossil fuel reserves are rapidly depleting, while energy consumption is increasing dramatically [1,2]. Until now, the most sustainable exploitation pathway for biowaste has been its conversion to biofuels; however, new applications are emerging. Coffee is one of the most widely consumed beverages in the world and is created from roasted beans of the plant genus Coffea and family Rubiaceae. On average, around 1 kg of soluble coffee can produce two kilograms of wet ground coffee [3]. As a result, large quantities of used coffee grounds from coffee shops are disposed of in landfills. Therefore, the disposal of coffee grounds accelerates the time required for the landfill to reach its capacity. Furthermore, this contributes to a global problem of food loss and waste, now estimated to be 2.1 billion tons of food wasted and a lost economic value of USD1.5 trillion globally by 2030 [4].

Hydrochar is a carbon-based material that is prepared by the hydrothermal carbonization of high moisture biomass waste, such as sewage sludge, algae or grass, in an aqueous environment at temperatures in the range of 180–260 °C [5]. Biochar is the solid product of biomass pyrolysis at temperatures in the range of 300–800 °C. The main advantage of hydrothermal carbonization over conventional pyrolysis is the potential to use wet biomasses as feedstock. Such carbonaceous materials prepared from spent coffee grounds have received much attention recently for their economic value and promising applications in environmental treatment technology. Although biochars exhibit higher surface areas and more extended porosities compared to hydrochars, the latter commonly have a higher number of oxygen-containing surface groups. Depending on the requirements, all these attributes are highly desirable for the development of functional materials such as catalysts or adsorbents. Various biochars and hydrochars have been used as substrates to disperse and stabilize nanoparticles (NPs) to enhance their reactivity for catalytic reactions [6,7,8].

Some examples of pollutants of concern today include heavy metals, herbicides, oil spills, pharmaceuticals and fertilizers. Compounds containing nitro groups have been determined in aqueous environments [9]. Due to the mutagenic and carcinogenic properties of nitro compounds, it is necessary to investigate their environmental fate as part of a strategy to prevent the contamination of receiving bodies. So far, various methods have been developed to remove nitro compounds from wastewater, including photochemical degradation, adsorption, microbial degradation, membrane distillation and electrocoagulation. However, these methods often have practical limitations, such as a low removal efficiency, cost inefficiency and the formation of harmful by-products. The catalytic reduction in nitro compounds to amino derivatives is an alternative and emerging process for the elimination of toxic nitro compounds from the environment. As a result of their unique and distinctive properties, nanomaterials have attracted great interest in recent years. In particular, transition metal/metal oxide nanocatalysts, with their unique physical and chemical properties, have attracted significant attention for their application in various fields [10,11,12]. The design and preparation of such catalysts has attracted a lot of attention for industrial processes, since they can be magnetically recovered after use, washed and reapplied [13].

In the framework of circular bio-economy, the rationale behind this work is to develop a novel pathway for the utilization of coffee waste and the production of a high added-value material. Therefore, spent coffee grounds were converted to hydrochar through hydrothermal carbonization. This process helps to increase the structural and chemical stability of the coffee grounds. The resultant hydrochar was then used as a substrate for the deposition of Fe_3_O_4_ particles, followed by the dispersion of Pd nanoparticles on the magnetic substrate surface. The chemical structure and composition of the nanocatalyst (referred to as Pd-Fe_3_O_4_-CWH thereof) were determined by various imaging and spectroscopic methods. Pd-Fe_3_O_4_-CWH was then applied as heterogeneous nanocatalyst for the reduction in 4-nitrobenzoic acid (4-NBA), 4-nitroaniline (4-NA), 4-nitro-o-phenylenediamine (4-NPD), 2-nitroaniline (2-NA) and 3-nitroanisole (3-NAS), using NaBH_4_ as a reducing reagent. The respective aniline products were determined by high performance liquid chromatography. A detailed investigation of the mechanism of reduction in the nitro groups was beyond the scope of this study. Finally, the reusability of the nanocatalyst was investigated by applying it in six successive catalytic runs.

## 2. Experimental Part

### 2.1. Materials and Methods

Spent coffee grounds were collected from a coffee shop. All nitro aromatic compounds, sodium borohydride (NaBH_4_, 99%), FeSO_4_·7H_2_O (4.2 g), FeCl_3_·6H_2_O, PdCl_2_, ethanol and methanol were purchased from Merck Chemical (Istanbul, Turkey). Hydrothermal carbonization was performed in a Berghoff Ins.-Heidolph MR Hei-standard reactor (Heidolph Instruments GmbH & Co. KG, Schwabach, Germany). Reductions in the nitro compounds were monitored by using a PerkinElmer Flexar Series HPLC system (Waltham, MA, USA). SEM images and EDS of CWH, Fe_3_O_4_–CWH and Pd-Fe_3_O_4_-CWH were recorded in a Supra 55 field emission (FE) microscope (ZEISS, Oberkochen, Germany). TEM images of Pd-Fe_3_O_4_-CWH were obtained in a JEOL JEM-1011 instrument. A SmartLab SE instrument Rigaku, Tokyo, Japan) was used to obtain the XRD patterns for the nanocatalyst. The exact Pd loading on Pd-Fe_3_O_4_-CWH was determined by inductively coupled plasma optical emission spectrometry (ICP-OES) (Thermo Scientific iCAP 6500, Manchester, UK).

### 2.2. Preparation and Characterization of Pd-Fe_3_O_4_-CWH Nanocatalyst

Hydrochar was prepared through hydrothermal carbonization at 200 °C and 2 h treatment time.

**Figure 1:**
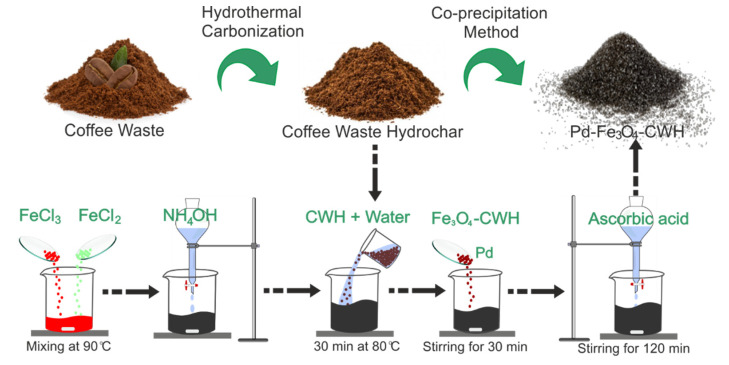
Schematic diagram of the Pd-Fe_3_O_4_-CWH catalyst preparation.

Fe_3_O_4_–CWH was obtained by the following procedure, discussed in detail in our previous study [5]. First, FeSO_4_·7H_2_O (4.2 g) and FeCl_3_·6H_2_O (6.1 g) were dissolved in 100 mL distilled water and heated to 90 °C. Ammonium hydroxide (10 mL-26%) and a suspension of 1 g of CWH in 200 mL of water were mixed, the mixture was stirred at 90 °C for 40 min and, finally, cooled to 25 °C. Fe_3_O_4_–CWH was collected as a black precipitate by filtering, being repeatedly washed with distilled water until a neutral pH was reached, dried at 70 °C for 18 h and stored. The next procedure was applied to load the Pd nanoparticles onto Fe_3_O_4_–CWH. A total of 0.25 g of Fe_3_O_4_–CWH was suspended in 30 mL water and a specific quantity of Na_2_PdCl_4_ (as the Pd precursor) was added, representative of a 5% Pd loading. After 40 min of stirring at 25 °C, an ascorbic acid solution (n_ascorbicacid_:n_Pd_ ¼ 2:1) was added and allowed to react for 130 min. After filtration, the solid catalyst was rinsed repeatedly with distilled water. Pd-Fe_3_O_4_-CWH was recovered with extremal magnet after drying at 80 °C for 12 h. The preparation of Pd-Fe_3_O_4_-CWH nanocatalyst is presented in Figure 1.

### 2.3. Reduction in Nitro Compounds to Anilines

For the reduction in the nitro compounds to the respective amino derivatives, 20 mg of Pd-Fe_3_O_4_-CWH was transferred into 1 mL of nitro compound (3 × 10^−4^ M), followed by stirring for 1 min at room temperature. Freshly prepared NaBH_4_ (0.08 M, 0.4 mL) was then added to the reaction medium and the nitro compound reduction was followed by HPLC. Finally, the nanocatalyst was removed from the reaction media by a magnetic bar and reactivated by washing with water before using it for subsequent runs. Kinetic studies were performed at 25 °C by using 4-NBA as the model substrate and an excess concentration of NaBH_4_.

### 2.4. HPLC Analysis

The analysis of the reduced nitro-aromatic compounds was performed by using a PerkinElmer Flexar Series HPLC system (Waltham, MA, USA). Separation was achieved on a ZORBAX SB Phenyl column (150 mm × 4.6 mm, 5 μm, Agilent Technologies, Santa Clara, CA, USA) maintained at 25 °C. The mobile phase used was 20/79 *v/v* acetonitrile/water, to which 1% acetic acid was added. The flow rate was set at 1.0 mL·min^–1^ and the injection volume at 10 μL. UV detection was set at 270 nm.

## 3. Results and Discussion

### 3.1. Characterization

**Figure 2:**
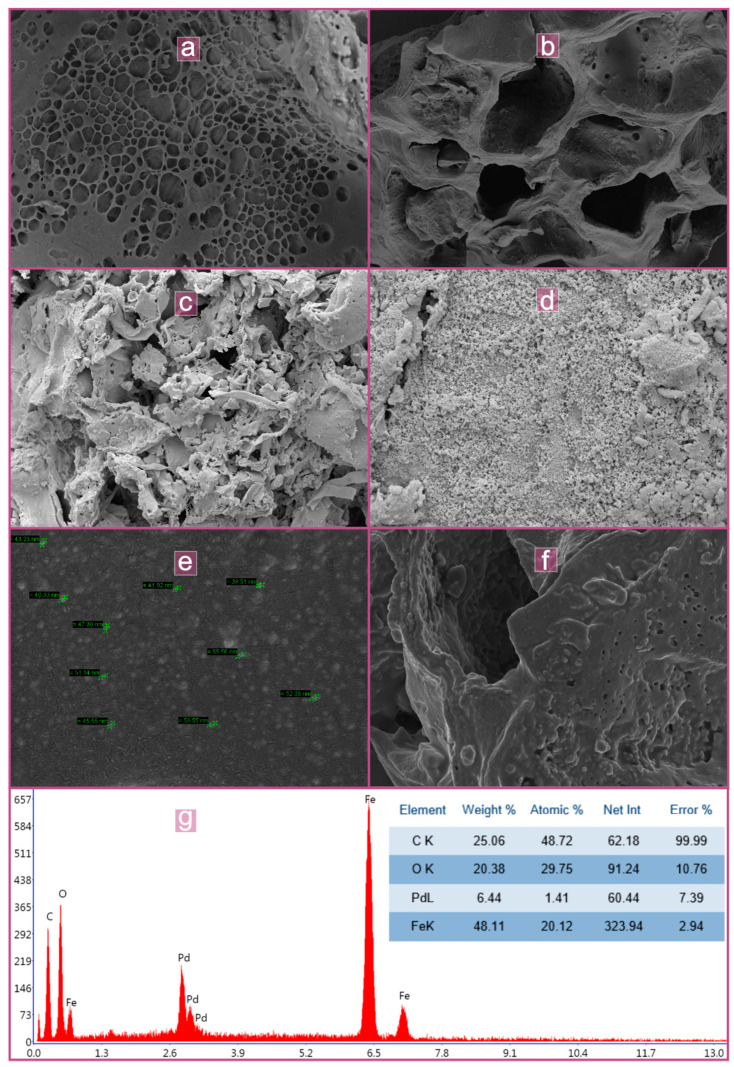
FE-SEM images of CWH (**a**,**b**), Fe_3_O_4_–CWH (**c**,**d**), Pd-Fe_3_O_4_-CWH nanocatalyst (**e**,**f**) and EDS spectrum of Pd-Fe_3_O_4_-CWH nanocatalyst (**g**).

Figure 2 depicts FE-SEM images and associated EDS data of Pd-Fe_3_O_4_-CWH. FE-SEM images of CWH showed an irregular but porous surface morphology (Figure 2a,b). Hydrochars are typically amorphous materials with a low degree of crystallinity [14,15]. Following the deposition of Fe_3_O_4_, it was observed that CWH’s surface morphology was not affected, but only covered by Fe_3_O_4_ particles (Figure 2c,d). The Fe_3_O_4_ cluster sizes varied widely, ranging from tenths to hundreds of nanometers, confirming earlier studies [16]. Figure 2e indicates that Pd nanoparticles, shown as small dots, were homogeneously dispersed on the surface of Fe_3_O_4_-CWH [17]. The presence of C, O, Pd and Fe peaks in the EDX spectrum of the nanocatalyst confirmed the successful fabrication of Pd nanoparticles on Fe_3_O_4_–CWH.

**Figure 3:**
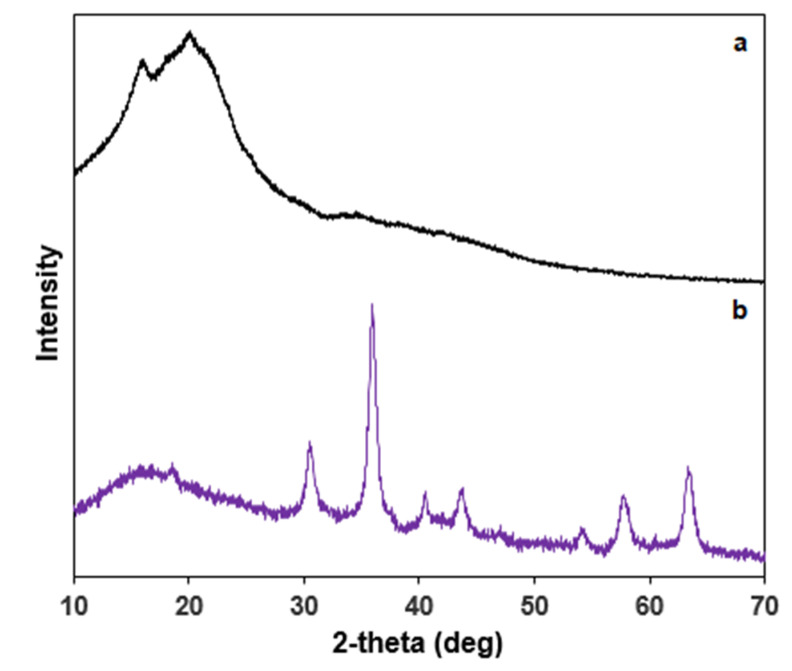
XRD diagram of (**a**) CWH and (**b**) Pd-Fe_3_O_4_-CWH nanocatalyst.

Figure 3 illustrates the XRD pattern of Pd-Fe_3_O_4_-CWH. The six sharp peaks at 30.16°, 35.62°, 43.34, 53.45°, 57.13° and 62.83° represent the Fe_3_O_4_ crystalline phases and correspond to the (220), (311), (400), (422), (511) and (440) planes [18] (Figure 3b). Additionally, the two diffraction peaks at 40.19° and 46.65°, assigned to the (111) and (200) planes of metallic Pd [19,20] were easily observed in the XRD pattern of the nanocatalyst (Figure 3b). These peaks further confirmed the presence of Pd NPs on the Fe_3_O_4_–CWH surface.

**Figure 4:**
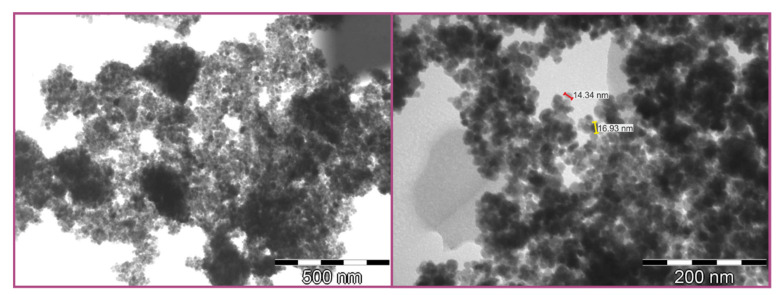
TEM images of Pd-Fe_3_O_4_-CWH nanocatalyst.

For a more detailed surface morphology, a TEM analysis of the Pd-Fe_3_O_4_-CWH nanocatalyst was performed and the corresponding images are displayed in Figure 4. The images highlighted the presence of Pd NPs as black dots, nearly homogenously grafted on Fe_3_O_4_-CWH.

**Figure 5:**
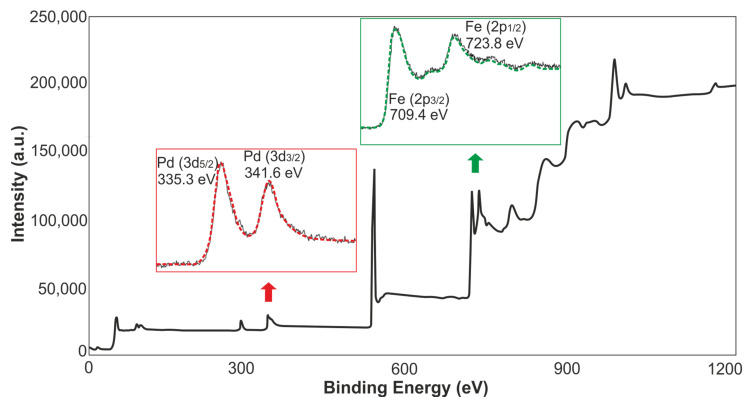
XPS analysis of Pd-Fe_3_O_4_-CWH nanocatalyst.

The XPS analysis (Figure 5) also confirmed the fabrication of the Pd-Fe_3_O_4_-CWH nanocatalyst by the display of Fe 2p (709.4 eV (2p_3/2_), 723.8 eV (2p_1/2_)) and Pd 3d (335.3 eV (3d_5/2_), 341.6 eV (3d_3/2_)) of metallic Pd(0), respectively, in the spectrum [21,22]. The two characteristic peaks of Pd(II), commonly seen at 338.0 and 343.6 eV in published works, were not observed [23]. This indicated the dominance of the Pd(0) speciation on our nanocatalyst.

### 3.2. Catalytic Activity Test

**Figure 6:**
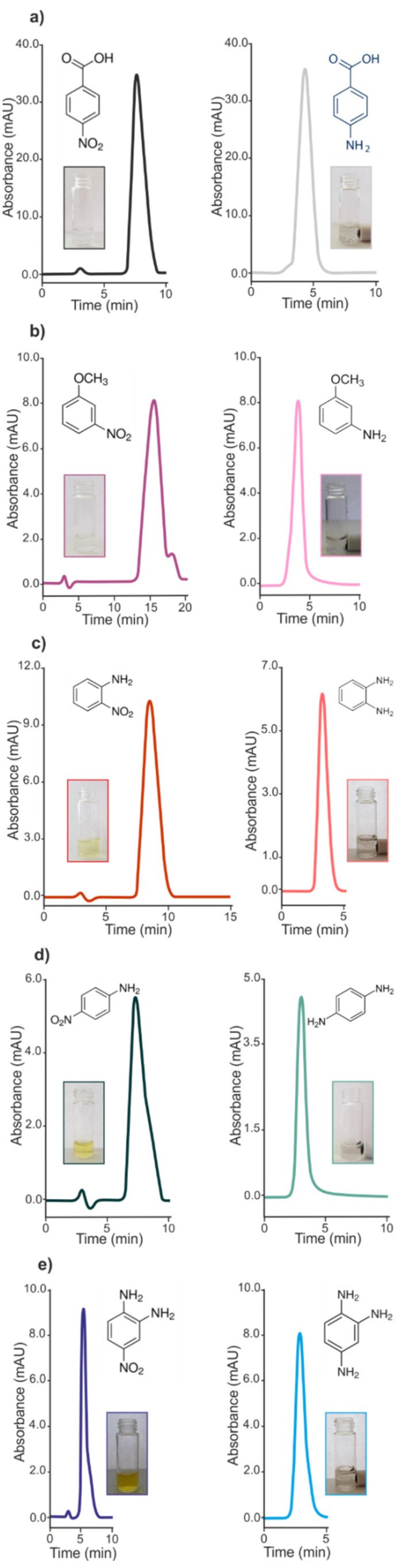
HPLC chromatograms of 4-nitrobenzoic acid (**a**), 3-nitroanisole (**b**), 2-nitroaniline (**c**), 4-nitroaniline (**d**), 4-nitro-o-phenylenediamine (**e**) and their reduction products.

**Scheme 1:**
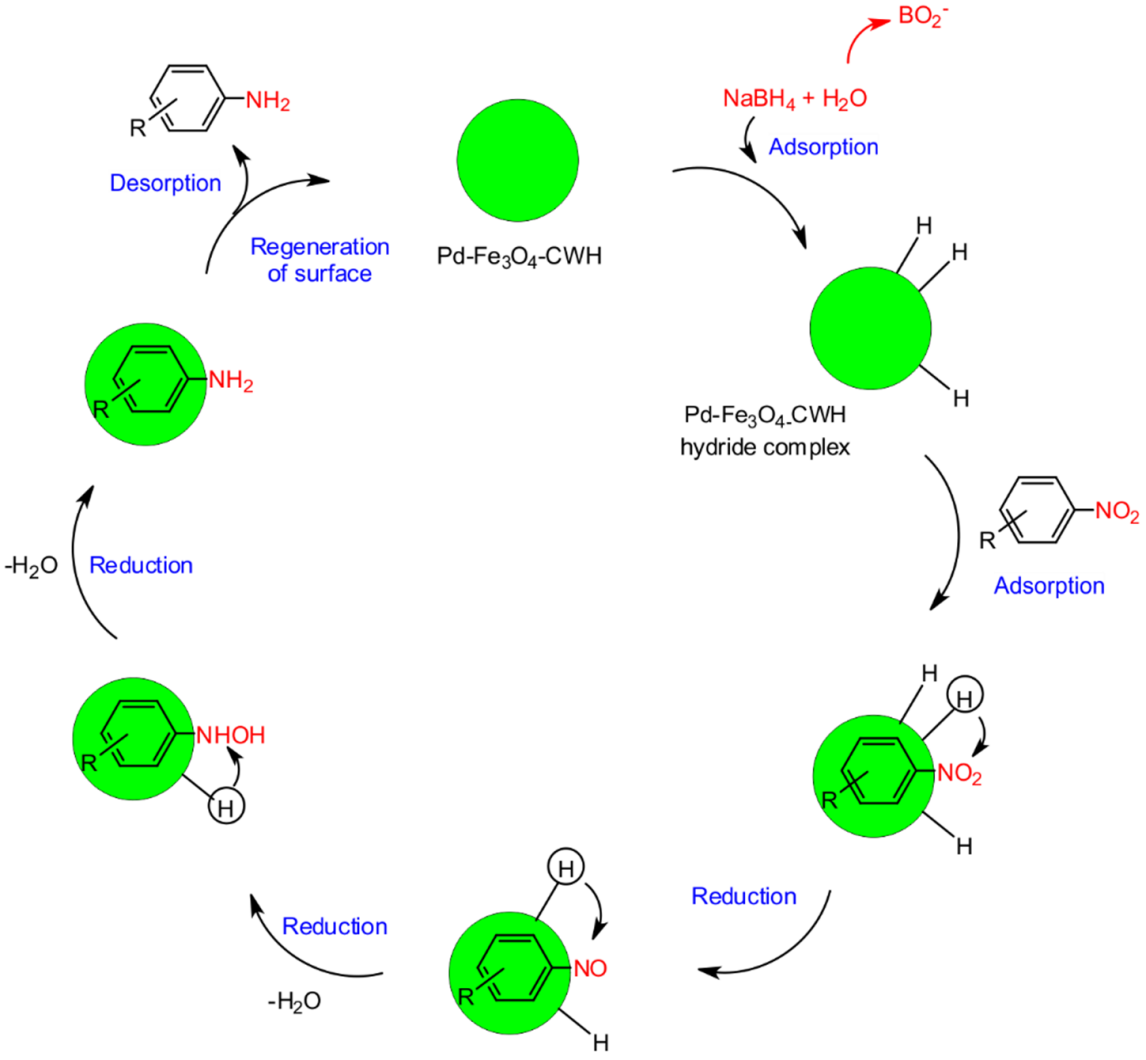
Proposed reaction mechanism for Pd-Fe_3_O_4_-CWH catalyzed reduction in nitroarenes.

The performance of the nanocatalyst was explored by employing it in the catalytic reduction in various nitro compounds in the presence of NaBH_4_ as a hydrogen source. To test the catalytic performance of Pd-Fe_3_O_4_-CWH and establish the optimal reaction conditions (amount of catalyst, concentration of NaBH_4_ and nitro compound) in the reduction in nitro compounds, 4-NA was selected as a representative substrate and control studies were performed (Table 1). The progress of catalytic reactions was followed by high performance liquid chromatography by monitoring the retention times of both substrates and reduced products (Figure 6). As seen in Table 1, the desired product was obtained in less time in the presence of 20 mg of catalyst, 3 × 10^−4^ M of nitro compound and 0.4 mL of NaBH4 (0.08 M). Subsequently, the catalytic behavior of Pd-Fe_3_O_4_-CWH was examined in the reduction in 2-NA, 4-NBA, 4-NPD and 3-NAS reductions with the detected optimal conditions and the obtained findings shown in Table 1. As seen in Table 2, the nitro compounds were successfully reduced to the corresponding aniline derivatives in very short times. For example, the Pd-Fe_3_O_4_-CWH nanocatalyst provided a complete reduction in 4-NBA within 60 s. Pd-Fe_3_O_4_-CWH catalyzed the 4-NA reduction within 82 s. Furthermore, the reduction in 2-NA proceeded quickly and was completed within 90 s. In a similar manner, the catalytic reductions in 4-NPD and 3-NAS were completed within 168 and 428 s, respectively. On the other hand, the reduction in 4-NA was performed without the Pd-Fe_3_O_4_-CWH catalyst, and no product formation was observed after 1 h. This result revealed the importance of the Fe_3_O_4_–CWH catalyst against the reduction in nitroarenes. Additionally, based on previous studies, the possible reaction mechanism for the Pd-Fe_3_O_4_-CWH catalyzed reduction in nitroarenes was given in Scheme 1 [24,25].

To indicate the superiority of Pd-Fe_3_O_4_-CWH over other reported catalysts, its catalytic performance for the reduction in 2-NA was compared to that of other catalysts reported in the literature. As seen in Table 3, our nanocatalyst compared favorably with respect to the reaction time for the complete reduction in 2-NA. Furthermore, some of the catalysts reported in the table required multiple, complex preparation steps, and used substrates from non-renewable sources.

For the kinetic study, a high excess of NaBH_4_ meant that the rate constant could be assumed to be independent of the NaBH_4_ concentration and a pseudo-first-order kinetics model could be applied to the reduction in 4-NBA [35]. The pseudo-first-order rate constant (*k*) value was calculated from the slope of the following equation:

(1) $$ln\frac{4-NBA_{t}}{4-NBA_{0}}=-kt$$

where 4-NBA_t_ and 4-NBA_0_ are the 4-NBA concentration at time *t* and initial concentration, respectively. As indicated by the regression coefficient (R^2^ = 0.9829), the reduction data fitted very well to the pseudo-first-order model (Figure S1). This observation agreed well with earlier studies, which examined the reduction of nitroarenes under the influence of various catalysts [36,37]. The rate constant was determined as 0.1479 min^−1^, indicating a kinetically unhindered process with no induction period, in contrast to some earlier studies that showed that Pd catalysts have an initial stage of reduced performance during the reduction in nitro compounds [38,39,40].

### 3.3. Recyclability of Pd-Fe_3_O_4_-CWH

**Figure 7:**
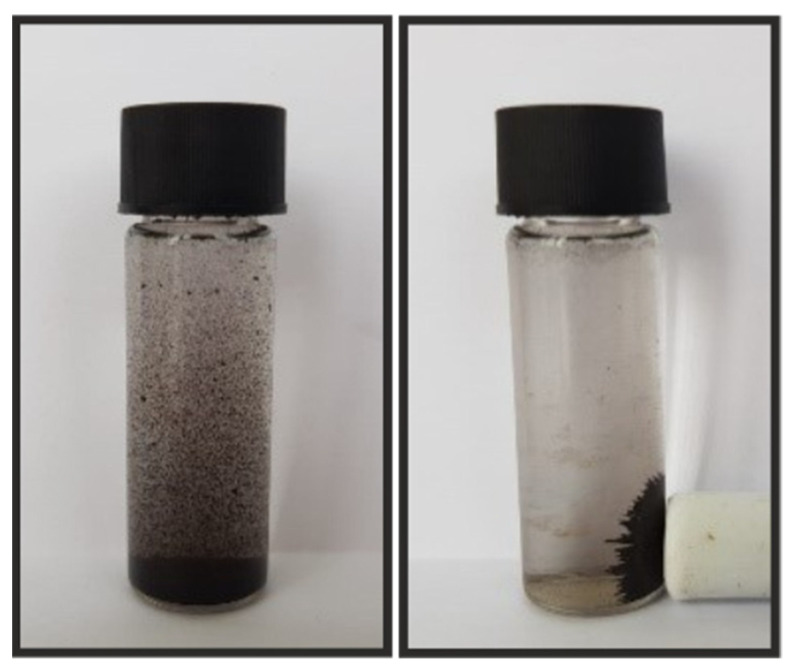
Recoverability of Fe_3_O_4_-CWH nanocatalyst with external magnet.

**Figure 8:**
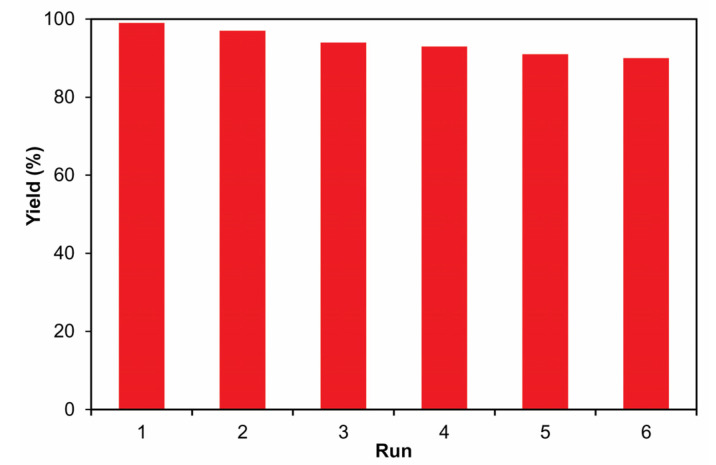
The recycling of the Pd-Fe_3_O_4_-CWH nanocatalyst.

One of the most important properties of heterogeneous catalysts is their reusability, a valuable attribute for industrial applications. Therefore, the recycling potential of Pd-Fe_3_O_4_-CWH was evaluated on 4-NBA reduction. The nanocatalyst was readily separated from reaction media magnetically after the 4-NBA reduction and rinsed with water to use in the further reactions (Figure 7). Reusability/recoverability studies showed that Pd-Fe_3_O_4_-CWH was reused up to a number six runs without any significant loss of catalytic performance (Figure 8).

## 4. Conclusions

It was demonstrated that coffee waste hydrochar can be used as a substrate for the development of an efficient nanocatalyst. Overall, our approach combined concepts of waste management, circular bio-economy and wastewater treatment. Therefore, biomass-based materials can provide sustainable building blocks for the production of catalysts for environmental remediation. Fe_3_O_4_ and Pd nanoparticles were deposited on the hydrochar surface through a simple process and the resultant catalyst exhibited a positive performance in the reduction in nitroarenes. Kinetically, the nanocatalyst showed no induction period, achieving complete reductions for a wide range of nitro compounds in very short times. Furthermore, the stability and reusability of the catalyst was established by collecting it through the use of a magnet and applying it in successive reaction runs. Further work should focus on testing the performance of the catalyst in the reduction in or oxidation reactions of other organic contaminants and investigating the economics of the process in detail, identifying necessary steps before scaling-up application.

## Figures and Tables

**Table 1:** The optimization of reaction parameters for 4-NA reduction.

4-NA (M)	NaBH_4_ (M)	Pd-Fe_3_O_4_-CWH (mg)	Reaction Time (s)
3 × 10^−4^	0.08	10	300
3 × 10^−4^	0.08	15	180
3 × 10^−4^	0.08	20	82
3 × 10^−4^	0.04	20	145
3 × 10^−4^	0.06	20	115
3.5 × 10^−4^	0.08	20	140
4 × 10^−4^	0.08	20	190

**Table 2:** The reduction and retention times of nitro compounds.

Substrate	Product	Reduction Time (s)	Retention Time (rt_min_) ^a^	Retention Time (rt_min_) ^b^
4-Nitrobenzoic Acid	4-Aminobenzoic acid	60	7.74	4.27
4-Nitroaniline	*4*-Phenylenediamine	82	7.33	2.95
2-Nitroaniline	*2*-Phenylenediamine	90	8.51	3.20
4-Nitro-o-phenylenediamine	1,2,4-Triaminobenzene	168	5.30	2.95
3-Nitroanisole	*3*-Anisidine	428	15.87	3.79

^a^ Retention time of substrates in HPLC analysis. ^b^ Retention time of products in HPLC analysis. Reduction conditions: nitro compounds (0.3 mM, 1.0 mL), NaBH_4_ (80 mM, 0.4 mL), Pd-Fe_3_O_4_-CWH catalyst (20 mg).

**Table 3:** Comparison of catalytic efficiency of Pd-Fe_3_O_4_-CWH with other reported catalysts in the 2-NA reduction.

Entry	Catalyst	Time	Ref.
1	Ag-PNA-BIS-2	8 h	[26]
2	Pd NPs/RGO	1.5 h	[27]
3	MMT@Fe_3_O_4_@Cu	6 min	[28]
4	Fe_3_O_4_-Glu-Ag	12 min	[29]
5	NiNPs/DNA	3 h	[30]
6	Cu–Acac@Am–Si–Fe_3_O_4_	5 min	[31]
7	SiO_2_@CuxO@TiO_2_	150 s	[32]
8	Ni@Au/KCC-1	660 s	[33]
9	Ag@CeO_2_ NCs	240 s	[34]
10	Pd-Fe_3_O_4_-CWH nanocatalyst	90 s	Present study

## Data Availability

The data presented in this study are available in Supplementary Materials.

## Supplementary Materials

The following are available online, Figure S1: Fitting of the 4-NBA reduction data to the pseudo first-order kinetic model.

## Sample Availability

Samples of the compounds are not available from the authors.
