# Supplementary material for: Preparation and Application of a Hydrochar-Based Palladium Nanocatalyst for the Reduction of Nitroarenes
Source: Molecules. 2021 Nov 13;26(22):6859. doi: 10.3390/molecules26226859 (PMC8621521; doi:10.3390/molecules26226859)
Supplement: Supplementary file 1 [file molecules-26-06859-s001.zip › molecules-1429196-supplementary.pdf]

Supplementary Material

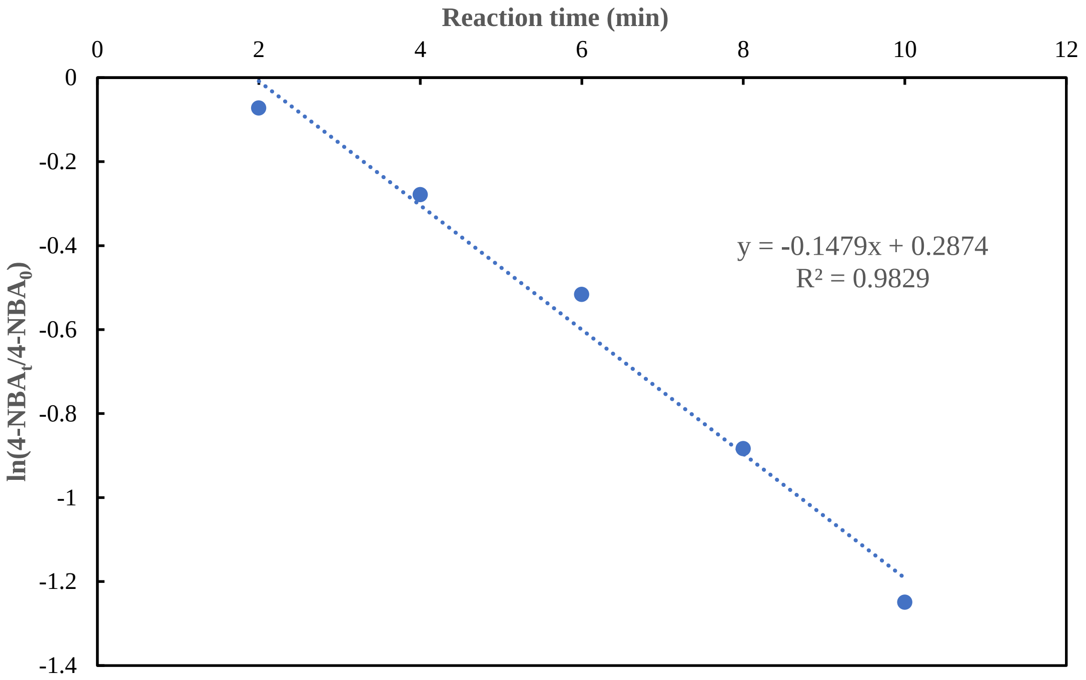

Figure S1. Fitting of the 4-NBA reduction data to the pseudo first-order kinetic model
